# Unraveling the immunosuppressive microenvironment of glioblastoma and advancements in treatment

**DOI:** 10.3389/fimmu.2025.1590781

**Published:** 2025-05-15

**Authors:** Dongxin Jiang, Yunqian Li

**Affiliations:** Department of Neurosurgery, The First Hospital of Jilin University, Changchun, China

**Keywords:** glioblastoma, immune microenvironment, cytokines, immunotherapy, immune checkpoint inhibitor, tumor vaccine

## Abstract

Glioblastoma, the most common and aggressive primary brain tumor, remains a significant challenge in oncology due to its immunosuppressive tumor microenvironment (TME). This review summarizes the complex interplay of immune cells and cytokines within the TME, which contribute to immune evasion and tumor progression. We further emphasize the synergistic crosstalk among these components and how it shapes therapeutic vulnerability. Besides, we highlight recent advancements in immunotherapy, including immune checkpoint inhibitors, CAR-T cell therapy, NK cell therapy, oncolytic viruses, and vaccine-based strategies. Despite promising preclinical and clinical results, overcoming the immunosuppressive TME remains a critical hurdle. This review underscores the potential of targeting the TME to enhance therapeutic outcomes in glioblastoma.

## 1 Introduction

Glioblastoma, the most common primary malignant brain tumor in the central nervous system (CNS), accounts for 80% of adult primary malignant brain tumors (1) and is the leading cause of intracranial malignancy-related deaths (2). Traditional treatments like surgical resection, radiotherapy, and temozolomide chemotherapy shows limited efficacy in improving the long-term survival rates of patients with glioblastoma (3–5). Emerging immunotherapies face challenges due to the immunosuppressive tumor immune microenvironment, a dynamic ecosystem crucial for tumor survival (6). The TME, comprising tumor-secreted cytokines, immune cells, and extracellular matrix, plays a pivotal role in tumor initiation, growth, invasion, and metastasis (7). Immune cells and cytokines in the TME not only facilitate immune evasion but also promote angiogenesis, proliferation, and invasiveness (8). This review focuses on the immune evasion mechanisms through immune cell infiltration and cytokines in the TME, and highlights the advancements in immunotherapy for glioblastoma.

## 2 Immune microenvironment of glioblastoma

### 2.1 Tumor-associated macrophages

Macrophages polarize into M1 (tumor-inhibiting) or M2 (tumor-promoting) phenotypes based on the microenvironment, with M1 TAMs enhancing Th1-mediated anti-tumor responses and counteracting immunosuppression (9). However, in advanced tumor stages, M2 TAMs dominate, suppressing adaptive immunity, promoting tumor growth, angiogenesis, and metastasis (10). Besides, hypoxia−driven lactate acts through GPR81-mediated signaling on TAMs to suppress NF-κB and YAP activation and cytokine production, thereby attenuating anti-tumor immunity (11, 12). In glioblastoma, M2 TAMs correlate with poor prognosis (13). Yu et al. (14) found TAM-derived CCL5 promotes glioblastoma cell migration and invasion, while Dong et al. (15) showed TAMs drive glioblastoma stem cell invasiveness via TREM1-mediated TGF-β2 secretion. These findings highlight TAMs’ critical role in the TME and potential therapeutic targets.

### 2.2 Tumor-infiltrating T lymphocytes

T cells are essential to the adaptive immune system, responding to antigens presented by dendritic cells and macrophages. They are categorized into CD4^+^ and CD8^+^ subsets based on surface markers and functions. CD4^+^ T cells recognize antigen-MHC class II complexes, initiating immune responses and activating other immune cells. Regulatory T cells (Tregs), a CD4^+^ subset expressing FOXP3, suppress pathological immune responses and maintain immune balance (16). CD8^+^ T cells, or cytotoxic T lymphocytes, directly kill infected cells via MHC class I interactions (17). In the TME, CD4^+^ T cells activate CD8^+^ T cells and NK cells, enhancing immune responses (18). They also secrete cytokines like IFN-γ and TNF-α, which have cytotoxic effects on tumors. Tregs maintain immune homeostasis by producing inhibitory cytokines (IL-10, IL-35, TGF-β), suppressing excessive immune activity, though their hyperactivity can impair anti-tumor immunity. CD8^+^ T cells recognize tumor antigens via TCRs, releasing perforin and granzyme to kill cancer cells and secreting IFN-γ and TNF-α to inhibit tumor growth (19). Glioblastomas reprogram T cells into dysfunctional or pro-tumor states, recruiting Tregs that secrete immunosuppressive cytokines (IL-10, TGF-β), suppressing CD8^+^ T cells and promoting glioblastoma survival (20). Loss of T cell anti-tumor function exacerbates immune evasion, aiding tumor progression.

### 2.3 Natural killer cells

NK cells, a lymphocyte subset in the innate immune system, exhibit cytotoxic capabilities crucial for tumor surveillance, with reduced activity linked to increased cancer risk. They target neoplastic cells via death receptor-mediated apoptosis and perforin/granzyme-mediated cytotoxicity, limiting primary tumor growth. However, glioblastomas show minimal NK cell infiltration. CRISPR-Cas9-mediated TIM3 knockout in NK cells enhances their cytotoxicity against glioblastoma cells (21). Additionally, NK cell-related genetic signatures predict glioblastoma malignancy and patient survival (22).

### 2.4 Dendritic cells

DCs are highly efficient antigen-presenting cells that play a central role in the immune system, linking innate and adaptive immune responses by activating other immune cells and promoting tumor-specific immunity (23). Upon exposure to pathogens, nucleic acids, or type I interferons, DCs undergo activation and maturation, acquiring the ability to effectively stimulate T cells (24). While the exact role of DCs in glioblastomas is still under investigation, current research highlights their interactions with tumor cells and the TME. Single cell RNA sequencing studies have identified conventional DC1 (cDC1), cDC2, and plasmacytoid DC subsets within glioblastoma specimens, each endowed with distinct transcriptional programs and functional potentials (25). Mature DCs up regulate co stimulatory molecules and secrete IL 12, fostering Th1 polarized anti-tumor responses (26). Conversely, glioblastoma-derived factors, like TGF β, IL-10, prostaglandin E_2_, can lock DCs in a tolerogenic state characterized by PD-L1 expression and diminished IL-12 production, thereby dampening T cell activation (27). A study by Friedrich et al. (28) indicated that DCs might contribute to the enhancement of anti-tumor immunity in glioblastomas, with their function potentially modulated by isocitrate dehydrogenase (IDH) mutations. These mutations may influence glioblastoma immune responses by altering the function of DCs.

### 2.5 Tumor-associated neutrophils

Neutrophils are actively involved in various stages of tumorigenesis, tumor progression, and metastasis, exhibiting a more intricate function than previously thought. These cells display both tumor-suppressive and tumor-promoting characteristics within the TME (29). They can directly kill tumor cells via reactive oxygen species (ROS) (30) or cell-cell contact (31), yet also support tumor growth by secreting immunosuppressive molecules like TGF-β, IL-6, and IL-8 (32). Neutrophil infiltration correlates with glioblastoma pathological grading (33, 34), and neutrophil extracellular traps (NETs) facilitate tumor cell migration and immune evasion (35). In glioblastomas, NET formation is driven by HMGB1 and the RAGE/ERK/NF-κB axis, which induces IL-8 release, promoting NETs (36).

### 2.6 Myeloid-derived suppressor cells

MDSCs, comprising granulocytic (G/PMN-MDSCs), monocytic (M-MDSCs), and early-stage (e-MDSCs) subsets, are immunosuppressive cells originating in the bone marrow (37). They mediate immune suppression through nitric oxide and cytokines, inhibiting cytotoxic T cells, NK cells, macrophages, and dendritic cells, thereby facilitating immune evasion (38). MDSCs also recruit Tregs, B cells, and M2 macrophages, potentially promoting glioblastoma progression (38). Elevated MDSC levels in glioblastoma patients’ peripheral blood correlate with tumor progression and survival, suggesting a disrupted immune environment and their potential as diagnostic and prognostic biomarkers (39).

### 2.7 B cells and microglia in glioblastoma

B cells constitute a minor proportion of immune cells within glioblastoma, yet they are pivotal in tumor progression and response to treatment (40). Within glioblastoma, the B cell population is predominantly composed of regulatory B cells, which exert immune-suppressive effects, and antigen-presenting B cells that facilitate T cell expansion (41, 42). These cells promote immune suppression and angiogenesis by secreting IL-10 and TGF-β, which inhibit T cell and NK cell activity, while also supporting brain development and tumor invasion (43). Furthermore, B cells release angiogenic factors, including VEGF, CXCL12, and CXCL13, which enhance neovascularization, ensuring the tumor’s access to essential nutrients and oxygen (44). Microglia is the principal immune cells in the CNS that maintains a quiescent state and exhibit a distinctive branched morphology under normal physiological conditions (45). When exposed to pathological stimuli, these cells become rapidly activated and undergo significant morphological changes to perform immune surveillance and defensive functions (46). In the context of the TME, microglia are attracted to the tumor site, guided by chemotactic factors like CCL2. They secrete a range of cytokines and growth factors, such as IL-6, TGF-β, and VEGF, which contribute to tumor progression by promoting metastasis and invasion (47).

### 2.8 Cross-talk between immune cells within the glioblastoma TME

The aforementioned immune subsets do not operate in isolation but engage in a highly coordinated network that ultimately dictates glioblastoma progression or regression. For example, TAMs release TGF-β and IL-10, inhibiting effector T cells and promoting Treg expansion, fostering immunosuppression (48). TAMs also suppress T cell function via PD-L1, exacerbating exhaustion and impairing anti-tumor immunity (49). The programmed cell death protein 1 (PD-1) and its ligand PD-L1 constitute a critical immune checkpoint mechanism that facilitates tumor immune escape. Malignant cells frequently overexpress PD-L1, which binds to PD-1 receptors on T lymphocytes, leading to T cell exhaustion and functional impairment (18). This immunosuppressive pathway is further amplified by multiple components of the tumor microenvironment, including: Immunosuppressive cytokines, TAMs, and Tregs. Besides, emerging studies demonstrate that crosstalk between gliomas and immune cells (including macrophages, neutrophils, dendritic cells, MDSCs, and NK cells facilitate oncogenic progression (50).

## 3 Cytokines in the immune microenvironment of glioblastoma

### 3.1 IL-10

IL-10, a key anti-inflammatory cytokine, modulates immune responses and prevents excessive inflammation (51). It is secreted by tumor cells, microglia, and astrocytes, not T or B cells (52). IL-10 deficiency releases pro-inflammatory cytokines, suppressing anti-tumor immunity and promoting growth (53), while high IL-10 levels may enhance tumor-specific immunity (54). Blocking IL-10 boosts anti-tumor immunity (55), and IL-10 may upregulate KPNA2, promoting tumor growth; KPNA2 knockout impairs these processes (56). *In vitro*, IL-10 enhances proliferation and invasion, while its blockade activates T cells (57). In glioblastoma, IL-10 promotes tumor proliferation and migration, with elevated levels correlating with malignancy (50–52), however, recent evidence indicates that IL 10 can paradoxically augment anti-tumor immunity by activating CD8^+^ T cells through the JAK1/STAT3 pathway, leading to enhanced granzyme B release and tumor lysis (58). Targeting TAMs to regulate IL-10 may enhance anti-tumor immunity, highlighting its therapeutic potential in glioblastoma.

### 3.2 IL-6

Research indicates IL-6 plays a critical role in tumorigenesis by promoting tumor cell proliferation, immune evasion, survival, angiogenesis, and metastasis (59). In glioblastoma, IL-6 is pivotal for immunosuppression, with elevated expression in tumor tissues correlating with disease progression and higher malignancy grades (60). Post-surgical reductions in IL-6 levels in serum and cerebrospinal fluid suggest its prognostic value for survival outcomes (61). Autocrine IL-6 secretion is linked to poor prognosis, driving tumor growth and invasion through: (1) direct stimulation of glioblastoma cell proliferation and survival; (2) STAT3 activation, which promotes tumor cell proliferation, inhibits apoptosis, and suppresses immune cell function; and (3) a cytokine feedback loop involving IL-6 and IL-10, sustaining tumor growth and impairing anti-tumor immunity (62), indicating IL-6 is a promising therapeutic target.

### 3.3 SDF-1

Chemokines regulate inflammation, immune responses, infection control, tissue damage, apoptosis, and cell migration. The SDF-1/CXCR4 axis, involving CXC chemokine ligand 12 (SDF-1) and receptor CXCR4, is critical for organ development (63). In glioblastomas, SDF-1 attracts stem cells to endothelial cells, where TGF-β induces pericyte differentiation, enhancing vascular activity and tumor growth (63, 64). Disrupting pericyte formation (e.g., ganciclovir) or inhibiting CXCR4 impairs tumor progression by limiting pericyte-endothelial integration (64). Elevated SDF-1 increases pericyte coverage, protecting vasculature and fostering resistance to anti-angiogenic therapies, contributing to recurrence (65).

### 3.4 TGF-β

TGF-β, a multifunctional regulatory polypeptide, is pivotal in cellular processes such as proliferation, apoptosis, differentiation, and immune surveillance (66). USP15 activates the TGF-β pathway, while its inhibition reduces TGF-β activity, suppressing Glioblastoma cell proliferation. TGF-β2 promotes autophagy via Smad-dependent and independent pathways, enhancing Glioblastoma invasion (67). pSMAD2, a key TGF-β signaling mediator, is found in the cytoplasm and nucleus, serving as a biomarker for pathway activation. Elevated pSMAD2 in glioblastoma correlates with increased invasiveness, therapy resistance, and poorer survival (68, 69). TGF-β2 overexpression is linked to higher tumor grades (70). Trabedersen, a TGF-β2 inhibitor, improved survival in a Phase II trial (71). Macromolecular TGF-β antagonists show greater selectivity and therapeutic potential than small molecules (72).

### 3.5 Colony-stimulating factors

CSFs are essential for macrophage development. High M-CSF in glioblastoma correlates with poor survival (73), promoting M2 polarization linked to higher tumor grade and worse prognosis (74). Inhibiting GM-CSFR slows tumor progression without reducing macrophages (75); without GM-CSFR, cytokines sustain macrophage survival but reduce immune suppression (76). CSF receptor inhibitors may modulate TAM phenotypes, improving prognosis, but resistance limits their efficacy (77, 78), requiring combination therapies.

### 3.6 Vascular endothelial growth factor

**Figure 1:**
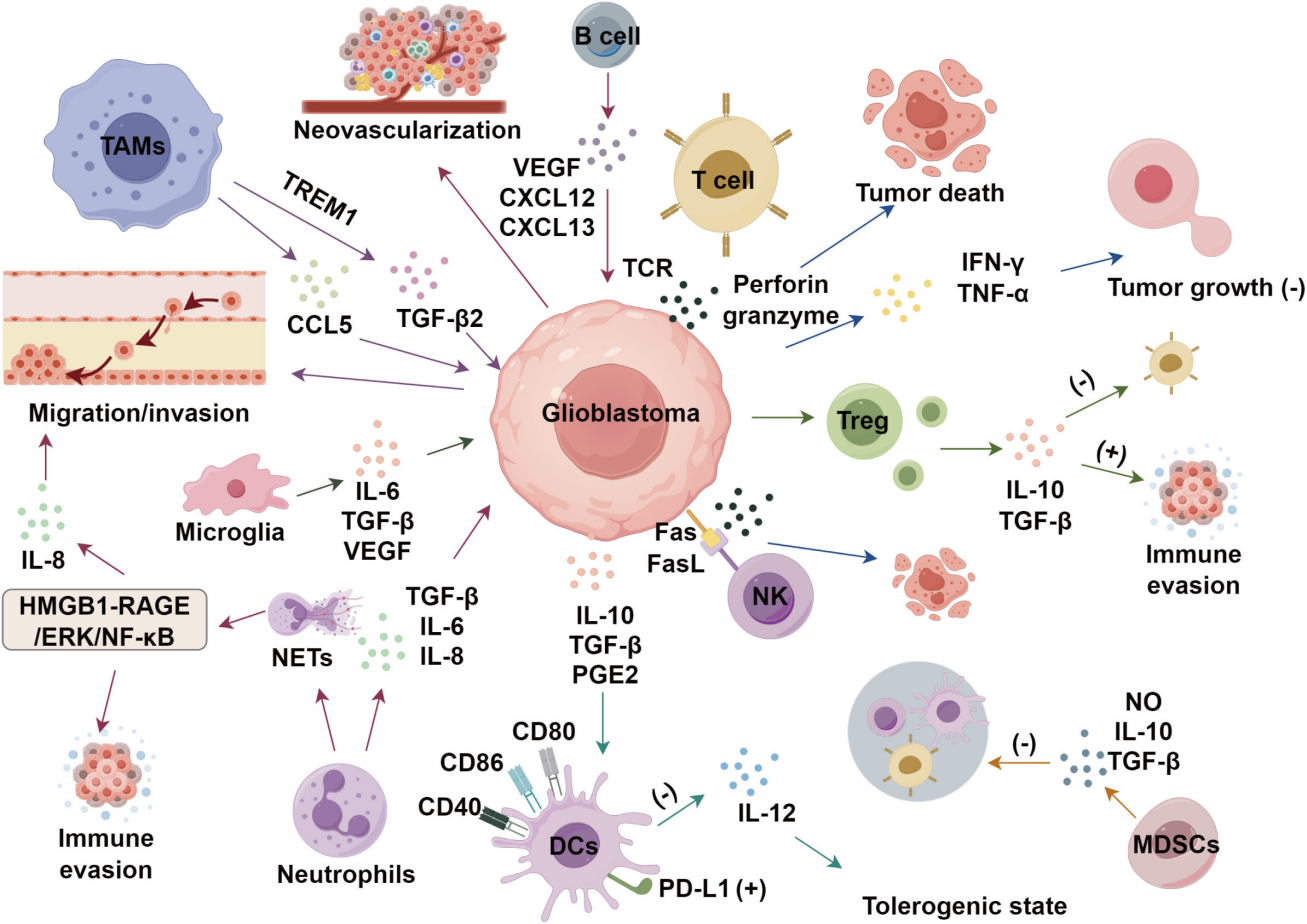
Immunosuppressive microenvironment of glioblastoma.

In glioblastoma progression, a critical aspect is neovascularization. VEGF plays a central role in this process, mediating paracrine and autocrine signals that activate receptor binding and subsequent signaling pathways, which foster the development of a new blood vessel network around the tumor. This promotes tumor growth and metastasis (79). Bevacizumab, a monoclonal antibody targeting VEGF-A, binds to circulating VEGF-A, thereby altering its interaction kinetics with endothelial cells and inhibiting angiogenesis (80). Clinical evidence indicates that bevacizumab therapy for glioblastoma can reduce tumor size, prolong progression-free survival, and diminish the reliance on corticosteroids to manage tumor-induced edema (81) (**Figure 1**).

## 4 Immunotherapy of glioblastoma

### 4.1 Immune checkpoint inhibitor therapy

Immune checkpoints regulate immune responses, preventing autoimmunity, but tumors exploit these mechanisms by expressing ligands, leading to T cell exhaustion and immune evasion (82–84). Immunotherapy utilizing immune checkpoint inhibitors (ICIs) has transformed solid tumor management by augmenting T cell-mediated antitumor responses. Among these, programmed death-1/programmed death-ligand 1 (PD-1/PD-L1) blockade has demonstrated clinical benefits across various malignancies, including gastrointestinal cancers (85, 86). In neuro-oncology, six active clinical trials are currently evaluating PD-1/PD-L1 targeting agents for glioblastoma treatment. Preliminary results from a Phase II investigation (NCT02968940) revealed improved outcomes when combining PD-L1 blockade with radiation therapy in recurrent cases, while pembrolizumab single-agent therapy extended median survival duration (87, 88). Nevertheless, subsequent Phase III evaluations, such as those conducted by Filley et al. (89) and trial NCT02617589, failed to demonstrate statistically significant survival advantages with nivolumab treatment, potentially attributable to the profoundly immunosuppressive characteristics of glioblastoma. Emerging combination approaches, particularly dual PD-1 and CTLA-4 inhibition (NCT03233152), represent promising therapeutic avenues (90). Indoleamine 2,3-dioxygenase (IDO), upregulated in glioblastomas, suppresses T cell function and correlates with poor prognosis (91–94). Preclinical studies support IDO inhibition (95). TIM-3, highly expressed in glioblastoma, enhances CD8^+^ T cell activity but correlates with aggressive tumors and worse prognoses (96, 97). These findings underscore the potential of targeting immune checkpoints in glioblastoma treatment.

### 4.2 CAR-T

Chimeric antigen receptors (CARs) are synthetic receptors designed to direct immune cells against tumor-associated antigens, enhancing anti-tumor responses (98). While CAR-T therapy has achieved FDA approval for CD19^+^ B-cell malignancies (99), its success in glioblastoma remains limited. Recent studies, however, indicate progress. Ahmed et al. (100) reported that HER2-targeted CAR-T cells were safe and feasible in GBM, though tumor suppression was modest. O’Rourke et al. (101) conducted the first clinical trial (NCT02209376) targeting EGFRvIII in recurrent GBM, showing CAR-T infiltration, reduced EGFRvIII expression, and TME modulation, despite no significant regression. Earlier data suggested persistent EGFRvIII in recurrent GBM (102), but subsequent trials (NCT02208362, NCT03389230) (103) confirmed that EGFRvIII-targeted CAR-T suppresses tumor activity. Key challenges include antigen loss, TME immunosuppression, and toxicity (104). A novel TanCAR strategy, combining IL-13 and EphA2scFv, improves GBM targeting while minimizing off-tumor effects, presenting a potential solution (105).

### 4.3 NK cell therapy

NK cells, crucial components of the innate immune system, directly target and eliminate tumor cells by secreting interferons, perforins, and granzymes, and upregulating death receptors like Fas ligand and TRAIL. They induce apoptosis via the caspase cascade and mediate antibody-dependent cellular cytotoxicity through FcγRIIIA/CD16A. NK cells also enhance T-cell-mediated tumor immunity by sustaining DC populations and promoting tumor antigen presentation (106). Besides, NK cells infiltrate glioblastomas more than T-cells (107). Clinical trials, such as Lim et al. (108), demonstrated NK cell therapy’s safety and efficacy in glioblastoma patients, with median OS of 22.5 months and PFS of 10 months. Shaim et al. (109) and Wang et al. (110) highlighted enhanced tumor suppression when NK cells were combined with integrin/TGF-β inhibitors or other therapies. However, challenges like *in vivo* NK cell persistence, limited cytokine support, and immunotherapy efficacy barriers must be addressed for broader clinical application (111).

### 4.4 Oncolytic virus

OVs, a promising immunotherapy, selectively target and replicate within tumor cells, destroying them while sparing healthy cells. Research includes adenovirus-based therapies and herpes simplex virus (HSV) variants, with notable preclinical success. In Japan, the modified HSV G47D is approved for glioblastoma treatment (112). Treatment with OV DNX-2401 in glioblastoma patients, tumor reduction, partial remission, and disease stabilization, with a median survival of 17.8 months were observed (113). Bernstock et al. (114) reported improved 2-year and 3-year survival rates with viral therapy. While preclinical studies confirm OV safety and efficacy, further clinical trials are needed to establish OVs as a standard glioblastoma treatment.

### 4.5 Tumor vaccine

Dendritic Cells (DCs) are pivotal in antitumor immunity, activating CD8^+^ and CD4^+^ T cells via MHC I/II presentation, driving lymphocyte proliferation and tumor antigen targeting (115). DC vaccines (DCVs) like Sipuleucel-T (116) demonstrate clinical potential. In glioblastoma, DCVax-L (NCT00045968) enhanced median survival without toxicity (117), while ICT-107 showed comparable efficacy (118). Limitations include suboptimal DC maturation, migration, complex manufacturing, antigen selection hurdles, and cost (119). Heat shock proteins (HSPs), ubiquitous molecular chaperones, augment antigen presentation and T-cell activation (120). Preclinical data reveal HSP vaccines with radiotherapy suppress glioblastoma growth (121). Clinical studies report improved survival post-surgery with autologous HSP vaccines (122), and HSPPC-96 (NCT02122822) extended survival in newly diagnosed patients (123). However, some trials associate HSP vaccines with worsened outcomes when combined with chemo/radiotherapy (124). IDH1 mutations define a glioblastoma subset. Murine studies demonstrate IDH1 R132H vaccines elicit IFN-γ-dependent T-cell responses, suppressing tumors (125). A study in glioma patients with IDH1 mutations found 93.3% developed immune responses, with 26/30 showing T-cell and 28/30 B-cell responses, confirming efficacy over 46.9 months median follow-up (126, 127). The NCT02454634 trial detected immune responses in IDH1 R132H^+^ gliomas but no survival benefit with adjuvant therapy (128).

## 5 Conclusion

Glioblastoma’s immunosuppressive TME, characterized by immune cell infiltration and cytokine-mediated immune evasion, plays a pivotal role in tumor progression and resistance to therapy. While traditional treatments have shown limited efficacy, emerging immunotherapies, such as immune checkpoint inhibitors, CAR-T cells, and oncolytic viruses, offer new hope. However, challenges like antigen escape, TME complexity, and treatment-related toxicity persist. Future research should focus on interdisciplinary collaboration and technological integration to elucidate glioblastoma regulatory networks, identify new targets, and refine personalized therapies. Combining cellular immunotherapy and molecular targeted therapy is a promising trend, offering hope for glioblastoma patients and insights for treating other solid tumors.

## References

[1] Stabellini N Krebs H Patil N Waite K Barnholtz-Sloan JS. Sex differences in time to treat and outcomes for gliomas. Front Oncol. (2021) 11:630597. doi: 10.3389/fonc.2021.630597 33680971 PMC7933512

[2] Ostrom QT Cioffi G Gittleman H Patil N Waite K Kruchko C. CBTRUS statistical report: primary brain and other central nervous system tumors diagnosed in the United States in 2012-2016. Neuro Oncol. (2019) 21:v1–v100. doi: 10.1093/neuonc/noz150 31675094 PMC6823730

[3] Rodriguez-Camacho A Flores-Vazquez JG Moscardini-Martelli J Torres-Rios JA Olmos-Guzman A Ortiz-Arce CS. Glioblastoma treatment: state-of-the-Art and future perspectives. Int J Mol Sci. (2022) 23:7207. doi: 10.3390/ijms23137207 35806212 PMC9267036

[4] Chen J Lin A Luo PJCPA. Advancing pharmaceutical research: A comprehensive review of cutting-edge tools and technologies. Curr Pharm Anal. (2024) 21:1–19. doi: 10.1016/j.cpan.2024.11.001

[5] Wang Z Zhao Y Zhang LJCPA. Emerging trends and hot topics in the application of multi-omics in drug discovery: A bibliometric and visualized study. Curr Pharm Anal. (2024) 21:20–32. doi: 10.1016/j.cpan.2024.12.001

[6] Shaul ME Fridlender ZG. Tumour-associated neutrophils in patients with cancer. Nat Rev Clin Oncol. (2019) 16:601–20. doi: 10.1038/s41571-019-0222-4 31160735

[7] Lei X Lei Y Li JK Du WX Li RG Yang J. Immune cells within the tumor microenvironment: Biological functions and roles in cancer immunotherapy. Cancer Lett. (2020) 470:126–33. doi: 10.1016/j.canlet.2019.11.009 31730903

[8] Berraondo P Sanmamed MF Ochoa MC Etxeberria I Aznar MA Perez-Gracia JL. Cytokines in clinical cancer immunotherapy. Br J Cancer. (2019) 120:6–15. doi: 10.1038/s41416-018-0328-y 30413827 PMC6325155

[9] Zhu C Kros JM Cheng C Mustafa D. The contribution of tumor-associated macrophages in glioma neo-angiogenesis and implications for anti-angiogenic strategies. Neuro Oncol. (2017) 19:1435–46. doi: 10.1093/neuonc/nox081 PMC5737221 28575312

[10] Liu J Lu J Wu L Zhang T Wu J Li L. Targeting tumor-associated macrophages: Novel insights into immunotherapy of skin cancer. J Adv Res. (2025) 67:231–52. doi: 10.1016/j.jare.2024.01.013 PMC11725115 38242529

[11] Tao H Zhong X Zeng A Song LJ. Unveiling the veil of lactate in tumor-associated macrophages: a successful strategy for immunometabolic therapy. Front Immunol. (2023) 14:1208870. doi: 10.3389/fimmu.2023.1208870 37564659 PMC10411982

[12] Yang K Xu J Fan M Tu F Wang X Ha T. Lactate suppresses macrophage pro-inflammatory response to LPS stimulation by inhibition of YAP and NF-kappaB activation via GPR81-mediated signaling. Front Immunol. (2020) 11:587913. doi: 10.3389/fimmu.2020.587913 33123172 PMC7573489

[13] Cai J Hu Y Ye Z Ye L Gao L Wang Y. Immunogenic cell death-related risk signature predicts prognosis and characterizes the tumour microenvironment in lower-grade glioma. Front Immunol. (2022) 13:1011757. doi: 10.3389/fimmu.2022.1011757 36325335 PMC9618960

[14] Yu-Ju Wu C Chen CH Lin CY Feng LY Lin YC Wei KC. CCL5 of glioma-associated microglia/macrophages regulates glioma migration and invasion via calcium-dependent matrix metalloproteinase 2. Neuro Oncol. (2020) 22:253–66. doi: 10.1093/neuonc/noz189 PMC7032635 31593589

[15] Dong M Zhang X Peng P Chen Z Zhang Y Wan L. Hypoxia-induced TREM1 promotes mesenchymal-like states of glioma stem cells via alternatively activating tumor-associated macrophages. Cancer Lett. (2024) 590:216801. doi: 10.1016/j.canlet.2024.216801 38479552

[16] Savage PA Klawon DEJ Miller CH. Regulatory T cell development. Annu Rev Immunol. (2020) 38:421–53. doi: 10.1146/annurev-immunol-100219-020937 31990619

[17] Waldman AD Fritz JM Lenardo MJ. A guide to cancer immunotherapy: from T cell basic science to clinical practice. Nat Rev Immunol. (2020) 20:651–68. doi: 10.1038/s41577-020-0306-5 PMC7238960 32433532

[18] Wang H Zhou H Xu J Lu Y Ji X Yao Y. Different T-cell subsets in glioblastoma multiforme and targeted immunotherapy. Cancer Lett. (2021) 496:134–43. doi: 10.1016/j.canlet.2020.09.028 33022290

[19] Durgeau A Virk Y Corgnac S Mami-Chouaib F. Recent advances in targeting CD8 T-cell immunity for more effective cancer immunotherapy. Front Immunol. (2018) 9:14. doi: 10.3389/fimmu.2018.00014 29403496 PMC5786548

[20] Liu S Zhang C Wang B Zhang H Qin G Li C. Regulatory T cells promote glioma cell stemness through TGF-beta-NF-kappaB-IL6-STAT3 signaling. Cancer Immunol Immunother. (2021) 70:2601–16. doi: 10.1007/s00262-021-02872-0 PMC8360896 33576874

[21] Morimoto T Nakazawa T Matsuda R Nishimura F Nakamura M Yamada S. CRISPR-cas9-mediated TIM3 knockout in human natural killer cells enhances growth inhibitory effects on human glioma cells. Int J Mol Sci. (2021) 22:3489. doi: 10.3390/ijms22073489 33800561 PMC8036491

[22] Li C Liu F Sun L Liu Z Zeng Y. Natural killer cell-related gene signature predicts Malignancy of glioma and the survival of patients. BMC Cancer. (2022) 22:230. doi: 10.1186/s12885-022-09230-y 35236310 PMC8892793

[23] Macri C Pang ES Patton T O’Keeffe M. Dendritic cell subsets. Semin Cell Dev Biol. (2018) 84:11–21. doi: 10.1016/j.semcdb.2017.12.009 29246859

[24] Banchereau J Steinman RM. Dendritic cells and the control of immunity. Nature. (1998) 392:245–52. doi: 10.1038/32588 9521319

[25] Carenza C. Dendritic cell subsets in the pathogenesis of High Grade Gliomas. (2021).

[26] Lipscomb MF. Masten BJJPr: Dendritic cells: immune regulators in health and disease. Physiol Rev. (2002) 82:97–130. doi: 10.1152/physrev.00023.2001 11773610

[27] Zheng Y Ma X Feng S Zhu H Chen X Yu X. Dendritic cell vaccine of gliomas: challenges from bench to bed. Front Immunol. (2023) 14:1259562. doi: 10.3389/fimmu.2023.1259562 37781367 PMC10536174

[28] Friedrich M Hahn M Michel J Sankowski R Kilian M Kehl N. Dysfunctional dendritic cells limit antigen-specific T cell response in glioma. Neuro Oncol. (2023) 25:263–76. doi: 10.1093/neuonc/noac138 PMC9925697 35609569

[29] Jaillon S Ponzetta A Di Mitri D Santoni A Bonecchi R Mantovani A. Neutrophil diversity and plasticity in tumour progression and therapy. Nat Rev Cancer. (2020) 20:485–503. doi: 10.1038/s41568-020-0281-y 32694624

[30] Chang Y Syahirah R Wang X Jin G Torregrosa-Allen S Elzey BD. Engineering chimeric antigen receptor neutrophils from human pluripotent stem cells for targeted cancer immunotherapy. Cell Rep. (2022) 40:111128. doi: 10.1016/j.celrep.2022.111128 35858579 PMC9327527

[31] Sionov RV Fainsod-Levi T Zelter T Polyansky L Pham CT Granot Z. Neutrophil cathepsin G and tumor cell RAGE facilitate neutrophil anti-tumor cytotoxicity. Oncoimmunology. (2019) 8:e1624129. doi: 10.1080/2162402X.2019.1624129 31428521 PMC6685517

[32] Masucci MT Minopoli M Carriero MV. Tumor associated neutrophils. Their role in tumorigenesis, metastasis, prognosis and therapy. Front Oncol. (2019) 9:1146. doi: 10.3389/fonc.2019.01146 31799175 PMC6874146

[33] Fossati G Ricevuti G Edwards SW Walker C Dalton A Rossi ML. Neutrophil infiltration into human gliomas. Acta Neuropathol. (1999) 98:349–54. doi: 10.1007/s004010051093 10502039

[34] Weng W Chen X Gong S Guo L Zhang X. Preoperative neutrophil-lymphocyte ratio correlated with glioma grading and glioblastoma survival. Neurol Res. (2018) 40:917–22. doi: 10.1080/01616412.2018.1497271 30074469

[35] Demkow U. Neutrophil extracellular traps (NETs) in cancer invasion, evasion and metastasis. Cancers (Basel). (2021) 13:4495. doi: 10.3390/cancers13174495 34503307 PMC8431228

[36] Zha C Meng X Li L Mi S Qian D Li Z. Neutrophil extracellular traps mediate the crosstalk between glioma progression and the tumor microenvironment via the HMGB1/RAGE/IL-8 axis. Cancer Biol Med. (2020) 17:154–68. doi: 10.20892/j.issn.2095-3941.2019.0353 PMC7142852 32296583

[37] Lin YJ Wu CY Wu JY Lim M. The role of myeloid cells in GBM immunosuppression. Front Immunol. (2022) 13:887781. doi: 10.3389/fimmu.2022.887781 35711434 PMC9192945

[38] Lakshmanachetty S Cruz-Cruz J Hoffmeyer E Cole AP Mitra SS. New insights into the multifaceted role of myeloid-derived suppressor cells (MDSCs) in high-grade gliomas: from metabolic reprograming, immunosuppression, and therapeutic resistance to current strategies for targeting MDSCs. Cells. (2021) 10:893. doi: 10.3390/cells10040893 33919732 PMC8070707

[39] Raychaudhuri B Rayman P Ireland J Ko J Rini B Borden EC. Myeloid-derived suppressor cell accumulation and function in patients with newly diagnosed glioblastoma. Neuro Oncol. (2011) 13:591–9. doi: 10.1093/neuonc/nor042 PMC3107102 21636707

[40] Jain RW Yong VW. B cells in central nervous system disease: diversity, locations and pathophysiology. Nat Rev Immunol. (2022) 22:513–24. doi: 10.1038/s41577-021-00652-6 PMC8667979 34903877

[41] Hou D Wan H Katz JL Wang S Castro BA Vazquez-Cervantes GI. Antigen-presenting B cells promote TCF-1(+) PD1(-) stem-like CD8(+) T-cell proliferation in glioblastoma. Front Immunol. (2023) 14:1295218. doi: 10.3389/fimmu.2023.1295218 38268923 PMC10806106

[42] Lee-Chang C Rashidi A Miska J Zhang P Pituch KC Hou D. Myeloid-Derived Suppressive Cells Promote B cell-Mediated Immunosuppression via Transfer of PD-L1 in Glioblastoma. Cancer Immunol Res. (2019) 7:1928–43. doi: 10.1158/2326-6066.CIR-19-0240 PMC6891201 31530559

[43] Wischnewski V Maas RR Aruffo PG Soukup K Galletti G Kornete M. Phenotypic diversity of T cells in human primary and metastatic brain tumors revealed by multiomic interrogation. Nat Cancer. (2023) 4:908–24. doi: 10.1038/s43018-023-00566-3 PMC10293012 37217652

[44] Shonka N Piao Y Gilbert M Yung A Chang S DeAngelis LM. Cytokines associated with toxicity in the treatment of recurrent glioblastoma with aflibercept. Target Oncol. (2013) 8:117–25. doi: 10.1007/s11523-013-0254-0 PMC4802008 23345034

[45] Vidal-Itriago A Radford RAW Aramideh JA Maurel C Scherer NM Don EK. Microglia morphophysiological diversity and its implications for the CNS. Front Immunol. (2022) 13:997786. doi: 10.3389/fimmu.2022.997786 36341385 PMC9627549

[46] Hanisch UK Kettenmann H. Microglia: active sensor and versatile effector cells in the normal and pathologic brain. Nat Neurosci. (2007) 10:1387–94. doi: 10.1038/nn1997 17965659

[47] Kioi M Vogel H Schultz G Hoffman RM Harsh GR Brown JM. Inhibition of vasculogenesis, but not angiogenesis, prevents the recurrence of glioblastoma after irradiation in mice. J Clin Invest. (2010) 120:694–705. doi: 10.1172/JCI40283 20179352 PMC2827954

[48] Lin H Liu C Hu A Zhang D Yang H Mao Y. Understanding the immunosuppressive microenvironment of glioma: mechanistic insights and clinical perspectives. J Hematol Oncol. (2024) 17:31. doi: 10.1186/s13045-024-01544-7 38720342 PMC11077829

[49] Zhang H Liu L Liu J Dang P Hu S Yuan W. Roles of tumor-associated macrophages in anti-PD-1/PD-L1 immunotherapy for solid cancers. Mol Cancer. (2023) 22:58. doi: 10.1186/s12943-023-01725-x 36941614 PMC10029244

[50] Elguindy M Young JS Mondal I Lu RO Ho WS. Glioma-immune cell crosstalk in tumor progression. Cancers (Basel). (2024) 16:308. doi: 10.3390/cancers16020308 38254796 PMC10813573

[51] Wang X Wong K Ouyang W Rutz S. Targeting IL-10 family cytokines for the treatment of human diseases. Cold Spring Harb Perspect Biol. (2019) 11:a028548. doi: 10.1101/cshperspect.a028548 29038121 PMC6360861

[52] Widodo SS Dinevska M Furst LM Stylli SS Mantamadiotis T. IL-10 in glioma. Br J Cancer. (2021) 125:1466–76. doi: 10.1038/s41416-021-01515-6 PMC8609023 34349251

[53] Acuner-Ozbabacan ES Engin BH Guven-Maiorov E Kuzu G Muratcioglu S Baspinar A. The structural network of Interleukin-10 and its implications in inflammation and cancer. BMC Genomics. (2014) 15 Suppl 4:S2. doi: 10.1186/1471-2164-15-S4-S2 PMC4083408 25056661

[54] Tanikawa T Wilke CM Kryczek I Chen GY Kao J Nunez G. Interleukin-10 ablation promotes tumor development, growth, and metastasis. Cancer Res. (2012) 72:420–9. doi: 10.1158/0008-5472.CAN-10-4627 PMC3261323 22123924

[55] Kim BG Joo HG Chung IS Chung HY Woo HJ Yun YS. Inhibition of interleukin-10 (IL-10) production from MOPC 315 tumor cells by IL-10 antisense oligodeoxynucleotides enhances cell-mediated immune responses. Cancer Immunol Immunother. (2000) 49:433–40. doi: 10.1007/s002620000123 PMC11036946 11043850

[56] Zhang Z Huang X Li J Fan H Yang F Zhang R. Interleukin 10 promotes growth and invasion of glioma cells by up-regulating KPNA 2 *in vitro* . J Cancer Res Ther. (2019) 15:927–32. doi: 10.4103/jcrt.JCRT_284_19 31436254

[57] Kostianovsky AM Maier LM Anderson RC Bruce JN Anderson DE. Astrocytic regulation of human monocytic/microglial activation. J Immunol. (2008) 181:5425–32. doi: 10.4049/jimmunol.181.8.5425 18832699

[58] Tibbs E Cao XJC. Emerging canonical and non-canonical roles of granzyme B in health and disease. Cancers. (2022) 14:1436. doi: 10.3390/cancers14061436 35326588 PMC8946077

[59] Hirano T. IL-6 in inflammation, autoimmunity and cancer. Int Immunol. (2021) 33:127–48. doi: 10.1093/intimm/dxaa078 PMC7799025 33337480

[60] Shan Y He X Song W Han D Niu J Wang J. Role of IL-6 in the invasiveness and prognosis of glioma. Int J Clin Exp Med. (2015) 8:9114–20. PMC4538008 26309566

[61] Feng Y Wang J Tan D Cheng P Wu A. Relationship between circulating inflammatory factors and glioma risk and prognosis: A meta-analysis. Cancer Med. (2019) 8:7454–68. doi: 10.1002/cam4.v8.17 PMC6885890 31599129

[62] Johnson DE O’Keefe RA Grandis JR. Targeting the IL-6/JAK/STAT3 signalling axis in cancer. Nat Rev Clin Oncol. (2018) 15:234–48. doi: 10.1038/nrclinonc.2018.8 PMC5858971 29405201

[63] Hughes CE Nibbs RJB. A guide to chemokines and their receptors. FEBS J. (2018) 285:2944–71. doi: 10.1111/febs.2018.285.issue-16 PMC6120486 29637711

[64] Cheng L Huang Z Zhou W Wu Q Donnola S Liu JK. Glioblastoma stem cells generate vascular pericytes to support vessel function and tumor growth. Cell. (2013) 153:139–52. doi: 10.1016/j.cell.2013.02.021 PMC3638263 23540695

[65] Batchelor TT Sorensen AG di Tomaso E Zhang WT Duda DG Cohen KS. AZD2171, a pan-VEGF receptor tyrosine kinase inhibitor, normalizes tumor vasculature and alleviates edema in glioblastoma patients. Cancer Cell. (2007) 11:83–95. doi: 10.1016/j.ccr.2006.11.021 17222792 PMC2748664

[66] Batlle E Massague J. Transforming growth factor-beta signaling in immunity and cancer. Immunity. (2019) 50:924–40. doi: 10.1016/j.immuni.2019.03.024 PMC7507121 30995507

[67] Eichhorn PJ Rodon L Gonzalez-Junca A Dirac A Gili M Martinez-Saez E. USP15 stabilizes TGF-beta receptor I and promotes oncogenesis through the activation of TGF-beta signaling in glioblastoma. Nat Med. (2012) 18:429–35. doi: 10.1038/nm.2619 22344298

[68] Buwaneka P Ralko A Gorai S Pham H Cho W. Phosphoinositide-binding activity of Smad2 is essential for its function in TGF-beta signaling. J Biol Chem. (2021) 297:101303. doi: 10.1016/j.jbc.2021.101303 34655614 PMC8567202

[69] Capper D von Deimling A Brandes AA Carpentier AF Kesari S Sepulveda-Sanchez JM. Biomarker and histopathology evaluation of patients with recurrent glioblastoma treated with galunisertib, lomustine, or the combination of galunisertib and lomustine. Int J Mol Sci. (2017) 18:995. doi: 10.3390/ijms18050995 28481241 PMC5454908

[70] Hau P Jachimczak P Schlaier J Bogdahn U. TGF-beta2 signaling in high-grade gliomas. Curr Pharm Biotechnol. (2011) 12:2150–7. doi: 10.2174/138920111798808347 21619538

[71] Bogdahn U Hau P Stockhammer G Venkataramana NK Mahapatra AK Suri A. Targeted therapy for high-grade glioma with the TGF-beta2 inhibitor trabedersen: results of a randomized and controlled phase IIb study. Neuro Oncol. (2011) 13:132–42. doi: 10.1093/neuonc/noq142 PMC3018908 20980335

[72] Kaminska B Cyranowski S. Recent advances in understanding mechanisms of TGF beta signaling and its role in glioma pathogenesis. Adv Exp Med Biol. (2020) 1202:179–201. doi: 10.1007/978-3-030-30651-9_9 32034714

[73] Zhang H Luo YB Wu W Zhang L Wang Z Dai Z. The molecular feature of macrophages in tumor immune microenvironment of glioma patients. Comput Struct Biotechnol J. (2021) 19:4603–18. doi: 10.1016/j.csbj.2021.08.019 PMC8383063 34471502

[74] Komohara Y Ohnishi K Kuratsu J Takeya M. Possible involvement of the M2 anti-inflammatory macrophage phenotype in growth of human gliomas. J Pathol. (2008) 216:15–24. doi: 10.1002/path.v216:1 18553315

[75] Pyonteck SM Akkari L Schuhmacher AJ Bowman RL Sevenich L Quail DF. CSF-1R inhibition alters macrophage polarization and blocks glioma progression. Nat Med. (2013) 19:1264–72. doi: 10.1038/nm.3337 PMC3840724 24056773

[76] Garris C Pittet MJ. Therapeutically reeducating macrophages to treat GBM. Nat Med. (2013) 19:1207–8. doi: 10.1038/nm.3355 24100977

[77] Barca C Foray C Hermann S Herrlinger U Remory I Laoui D. The colony stimulating factor-1 receptor (CSF-1R)-mediated regulation of microglia/macrophages as a target for neurological disorders (Glioma, stroke). Front Immunol. (2021) 12:787307. doi: 10.3389/fimmu.2021.787307 34950148 PMC8688767

[78] Shankarappa PS Peer CJ Odabas A McCully CL Garcia RC Figg WD. Cerebrospinal fluid penetration of the colony-stimulating factor-1 receptor (CSF-1R) inhibitor, pexidartinib. Cancer Chemother Pharmacol. (2020) 85:1003–7. doi: 10.1007/s00280-020-04071-7 PMC8459199 32306101

[79] Long Y Tao H Karachi A Grippin AJ Jin L Chang YE. : dysregulation of glutamate transport enhances treg function that promotes VEGF blockade resistance in glioblastoma. Cancer Res. (2020) 80:499–509. doi: 10.1158/0008-5472.CAN-19-1577 31723000

[80] Garcia J Hurwitz HI Sandler AB Miles D Coleman RL Deurloo R. Bevacizumab (Avastin(R)) in cancer treatment: A review of 15 years of clinical experience and future outlook. Cancer Treat Rev. (2020) 86:102017. doi: 10.1016/j.ctrv.2020.102017 32335505

[81] Wefel JS Armstrong TS Pugh SL Gilbert MR Wendland MM Brachman DG. Neurocognitive, symptom, and health-related quality of life outcomes of a randomized trial of bevacizumab for newly diagnosed glioblastoma (NRG/RTOG 0825). Neuro Oncol. (2021) 23:1125–38. doi: 10.1093/neuonc/noab011 PMC8661434 33515019

[82] Mejia-Guarnizo LV Monroy-Camacho PS Turizo-Smith AD Rodriguez-Garcia JA. The role of immune checkpoints in antitumor response: a potential antitumor immunotherapy. Front Immunol. (2023) 14:1298571. doi: 10.3389/fimmu.2023.1298571 38162657 PMC10757365

[83] Sensi B Angelico R Toti L Conte L Coppola A Tisone G. Mechanism, potential, and concerns of immunotherapy for hepatocellular carcinoma and liver transplantation. Curr Mol Pharmacol. (2024) 17:e18761429310703. doi: 10.2174/0118761429310703240823045808 39225204

[84] Lin A Jiang A Huang L Li Y Zhang C Zhu L. : From chaos to order: optimizing fecal microbiota transplantation for enhanced immune checkpoint inhibitors efficacy. Gut Microbes. (2025) 17:2452277. doi: 10.1080/19490976.2025.2452277 39826104 PMC12716052

[85] Zhang Y Zhang Z. The history and advances in cancer immunotherapy: understanding the characteristics of tumor-infiltrating immune cells and their therapeutic implications. Cell Mol Immunol. (2020) 17:807–21. doi: 10.1038/s41423-020-0488-6 PMC7395159 32612154

[86] Lee EJ Yang JH Yang HJ Cho CK Choi JG Chung HS. Antitumor effect of korean red ginseng through blockade of PD-1/PD-L1 interaction in a humanized PD-L1 knock-in MC38 cancer mouse model. Int J Mol Sci. (2023) 24:1894. doi: 10.3390/ijms24031894 36768213 PMC9915403

[87] Richardson LG Miller JJ Kitagawa Y Wakimoto H Choi BD Curry WT. Implications of IDH mutations on immunotherapeutic strategies for Malignant glioma. Neurosurg Focus. (2022) 52:E6. doi: 10.3171/2021.11.FOCUS21604 35104795

[88] Cloughesy TF Mochizuki AY Orpilla JR Hugo W Lee AH Davidson TB. : Neoadjuvant anti-PD-1 immunotherapy promotes a survival benefit with intratumoral and systemic immune responses in recurrent glioblastoma. Nat Med. (2019) 25:477–86. doi: 10.1038/s41591-018-0337-7 PMC6408961 30742122

[89] Filley AC Henriquez M Dey M. Recurrent glioma clinical trial, CheckMate-143: the game is not over yet. Oncotarget. (2017) 8:91779–94. doi: 10.18632/oncotarget.21586 PMC5710964 29207684

[90] Duerinck J Schwarze JK Awada G Tijtgat J Vaeyens F Bertels C. : Intracerebral administration of CTLA-4 and PD-1 immune checkpoint blocking monoclonal antibodies in patients with recurrent glioblastoma: a phase I clinical trial. J Immunother Cancer. (2021) 9;e002296. doi: 10.1136/jitc-2020-002296 34168003 PMC8231061

[91] Wainwright DA Balyasnikova IV Chang AL Ahmed AU Moon KS Auffinger B. IDO expression in brain tumors increases the recruitment of regulatory T cells and negatively impacts survival. Clin Cancer Res. (2012) 18:6110–21. doi: 10.1158/1078-0432.CCR-12-2130 PMC3500434 22932670

[92] Zhai L Lauing KL Chang AL Dey M Qian J Cheng Y. The role of IDO in brain tumor immunotherapy. J Neurooncol. (2015) 123:395–403. doi: 10.1007/s11060-014-1687-8 25519303 PMC4641522

[93] O’Connor JC Lawson MA Andre C Briley EM Szegedi SS Lestage J. Induction of IDO by bacille Calmette-Guerin is responsible for development of murine depressive-like behavior. J Immunol. (2009) 182:3202–12. doi: 10.4049/jimmunol.0802722 PMC2664258 19234218

[94] Uyttenhove C Pilotte L Theate I Stroobant V Colau D Parmentier N. Evidence for a tumoral immune resistance mechanism based on tryptophan degradation by indoleamine 2,3-dioxygenase. Nat Med. (2003) 9:1269–74. doi: 10.1038/nm934 14502282

[95] Hanihara M Kawataki T Oh-Oka K Mitsuka K Nakao A Kinouchi H. Synergistic antitumor effect with indoleamine 2,3-dioxygenase inhibition and temozolomide in a murine glioma model. J Neurosurg. (2016) 124:1594–601. doi: 10.3171/2015.5.JNS141901 26636389

[96] Han S Feng S Xu L Shi W Wang X Wang H. Tim-3 on peripheral CD4(+) and CD8(+) T cells is involved in the development of glioma. DNA Cell Biol. (2014) 33:245–50. doi: 10.1089/dna.2013.2306 24512143

[97] Li G Wang Z Zhang C Liu X Cai J Wang Z. : Molecular and clinical characterization of TIM-3 in glioma through 1,024 samples. Oncoimmunology. (2017) 6:e1328339. doi: 10.1080/2162402X.2017.1328339 28919992 PMC5593703

[98] Sterner RC Sterner RM. CAR-T cell therapy: current limitations and potential strategies. Blood Cancer J. (2021) 11:69. doi: 10.1038/s41408-021-00459-7 33824268 PMC8024391

[99] Chong EA Levine BL Grupp SA Davis M Siegel DL Maude SL. CD19-Directed CAR T-cell (CTL019) product viability and clinical outcomes in Non-Hodgkin lymphomas and B-cell acute lymphoblastic leukemia. Blood. (2018) 132:197. doi: 10.1182/blood-2018-197 29784641

[100] Ahmed N Brawley V Hegde M Bielamowicz K Kalra M Landi D. : HER2-specific chimeric antigen receptor-modified virus-specific T cells for progressive glioblastoma: A phase 1 dose-escalation trial. JAMA Oncol. (2017) 3:1094–101. doi: 10.1001/jamaoncol.2017.0184 PMC5747970 28426845

[101] O’Rourke DM Nasrallah MP Desai A Melenhorst JJ Mansfield K Morrissette JJD. A single dose of peripherally infused EGFRvIII-directed CAR T cells mediates antigen loss and induces adaptive resistance in patients with recurrent glioblastoma. Sci Transl Med. (2017) 9:eaaa0984. doi: 10.1126/scitranslmed.aaa0984 28724573 PMC5762203

[102] Del Vecchio CA Giacomini CP Vogel H Jensen KC Florio T Merlo A. EGFRvIII gene rearrangement is an early event in glioblastoma tumorigenesis and expression defines a hierarchy modulated by epigenetic mechanisms. Oncogene. (2013) 32:2670–81. doi: 10.1038/onc.2012.280 22797070

[103] Brown CE Aguilar B Starr R Yang X Chang WC Weng L. : optimization of IL13Ralpha2-targeted chimeric antigen receptor T cells for improved anti-tumor efficacy against glioblastoma. Mol Ther. (2018) 26:31–44. doi: 10.1016/j.ymthe.2017.10.002 29103912 PMC5763077

[104] Bagley SJ Desai AS Linette GP June CH O’Rourke DM. CAR T-cell therapy for glioblastoma: recent clinical advances and future challenges. Neuro Oncol. (2018) 20:1429–38. doi: 10.1093/neuonc/noy032 PMC6176794 29509936

[105] Muhammad N Wang R Li W Zhang Z Chang Y Hu Y. A novel TanCAR targeting IL13Ralpha2 and EphA2 for enhanced glioblastoma therapy. Mol Ther Oncolytics. (2022) 24:729–41. doi: 10.1016/j.omto.2022.02.012 PMC8908045 35317513

[106] Burger MC Zhang C Harter PN Romanski A Strassheimer F Senft C. CAR-engineered NK cells for the treatment of glioblastoma: turning innate effectors into precision tools for cancer immunotherapy. Front Immunol. (2019) 10:2683. doi: 10.3389/fimmu.2019.02683 31798595 PMC6868035

[107] Cozar B Greppi M Carpentier S Narni-Mancinelli E Chiossone L Vivier E. Tumor-infiltrating natural killer cells. Cancer Discov. (2021) 11:34–44. doi: 10.1158/2159-8290.CD-20-0655 33277307 PMC7611243

[108] Lim J Park Y Ahn JW Sim J Kang SJ Hwang S. : Autologous adoptive immune-cell therapy elicited a durable response with enhanced immune reaction signatures in patients with recurrent glioblastoma: An open label, phase I/IIa trial. PloS One. (2021) 16:e0247293. doi: 10.1371/journal.pone.0247293 33690665 PMC7946298

[109] Shaim H Shanley M Basar R Daher M Gumin J Zamler DB. : Targeting the alphav integrin/TGF-beta axis improves natural killer cell function against glioblastoma stem cells. J Clin Invest. (2021) 131:e142116. doi: 10.1172/JCI142116 34138753 PMC8279586

[110] Wang J Toregrosa-Allen S Elzey BD Utturkar S Lanman NA Bernal-Crespo V. Multispecific targeting of glioblastoma with tumor microenvironment-responsive multifunctional engineered NK cells. Proc Natl Acad Sci U S A. (2021) 118;e2107507118. doi: 10.1073/pnas.2107507118 34740973 PMC8609337

[111] Pan C Zhai Y Li G Jiang T Zhang W. NK cell-based immunotherapy and therapeutic perspective in gliomas. Front Oncol. (2021) 11:751183. doi: 10.3389/fonc.2021.751183 34765554 PMC8576093

[112] Liu P Wang Y Wang Y Kong Z Chen W Li J. Effects of oncolytic viruses and viral vectors on immunity in glioblastoma. Gene Ther. (2022) 29:115–26. doi: 10.1038/s41434-020-00207-9 33191399

[113] Gallego Perez-Larraya J Garcia-Moure M Labiano S Patino-Garcia A Dobbs J Gonzalez-Huarriz M. : oncolytic DNX-2401 virus for pediatric diffuse intrinsic pontine glioma. N Engl J Med. (2022) 386:2471–81. doi: 10.1056/NEJMoa2202028 35767439

[114] Bernstock JD Blitz S Kang KD Friedman GK. Intraventricular immunovirotherapy; a translational step forward. Oncotarget. (2023) 14:40–3. doi: 10.18632/oncotarget.28343 PMC9836381 36634220

[115] Zhao T Li C Ge H Lin Y Kang D. Glioblastoma vaccine tumor therapy research progress. Chin Neurosurg J. (2022) 8:2. doi: 10.1186/s41016-021-00269-7 35045874 PMC8766628

[116] Gardner A de Mingo Pulido A Ruffell B. Dendritic cells and their role in immunotherapy. Front Immunol. (2020) 11:924. doi: 10.3389/fimmu.2020.00924 32508825 PMC7253577

[117] Liau LM Ashkan K Tran DD Campian JL Trusheim JE Cobbs CS. : First results on survival from a large Phase 3 clinical trial of an autologous dendritic cell vaccine in newly diagnosed glioblastoma. J Transl Med. (2018) 16:142. doi: 10.1186/s12967-018-1507-6 29843811 PMC5975654

[118] Wen PY Reardon DA Armstrong TS Phuphanich S Aiken RD Landolfi JC. : A randomized double-blind placebo-controlled phase II trial of dendritic cell vaccine ICT-107 in newly diagnosed patients with glioblastoma. Clin Cancer Res. (2019) 25:5799–807. doi: 10.1158/1078-0432.CCR-19-0261 PMC8132111 31320597

[119] Shemesh CS Hsu JC Hosseini I Shen BQ Rotte A Twomey P. Personalized cancer vaccines: clinical landscape, challenges, and opportunities. Mol Ther. (2021) 29:555–70. doi: 10.1016/j.ymthe.2020.09.038 PMC7854282 33038322

[120] Kelly M McNeel D Fisch P Malkovsky M. Immunological considerations underlying heat shock protein-mediated cancer vaccine strategies. Immunol Lett. (2018) 193:1–10. doi: 10.1016/j.imlet.2017.11.001 29129721

[121] Xiu Z Sun T Yang Y He Y Yang S Xue X. Curcumin enhanced ionizing radiation-induced immunogenic cell death in glioma cells through endoplasmic reticulum stress signaling pathways. Oxid Med Cell Longev. (2022) 2022:5424411. doi: 10.1155/2022/5424411 36238646 PMC9553401

[122] Bloch O Lim M Sughrue ME Komotar RJ Abrahams JM O’Rourke DM. Autologous heat shock protein peptide vaccination for newly diagnosed glioblastoma: impact of peripheral PD-L1 expression on response to therapy. Clin Cancer Res. (2017) 23:3575–84. doi: 10.1158/1078-0432.CCR-16-1369 PMC5511566 28193626

[123] Ji N Zhang Y Liu Y Xie J Wang Y Hao S. Heat shock protein peptide complex-96 vaccination for newly diagnosed glioblastoma: a phase I, single-arm trial. JCI Insight. (2018) 3:e99145. doi: 10.1172/jci.insight.99145 29769450 PMC6012501

[124] Alcaide-Leon P Luks TL Lafontaine M Lupo JM Okada H Clarke JL. Treatment-induced lesions in newly diagnosed glioblastoma patients undergoing chemoradiotherapy and heat-shock protein vaccine therapy. J Neurooncol. (2020) 146:71–8. doi: 10.1007/s11060-019-03336-3 PMC6939141 31728884

[125] Huang Y Wang Y Huang Z. A specific peptide vaccine against IDH1(R132H) glioma. Neurosci Bull. (2022) 38:223–5. doi: 10.1007/s12264-021-00791-9 PMC8821749 34739683

[126] Schumacher T Bunse L Pusch S Sahm F Wiestler B Quandt J. : A vaccine targeting mutant IDH1 induces antitumour immunity. Nature. (2014) 512:324–7. doi: 10.1038/nature13387 25043048

[127] Persico P Lorenzi E Losurdo A Dipasquale A Di Muzio A Navarria P. Precision oncology in lower-grade gliomas: promises and pitfalls of therapeutic strategies targeting IDH-mutations. Cancers (Basel). (2022) 14;1125. doi: 10.3390/cancers14051125 35267433 PMC8909346

[128] Platten M Bunse L Wick A Bunse T Le Cornet L Harting I. : A vaccine targeting mutant IDH1 in newly diagnosed glioma. Nature. (2021) 592:463–8. doi: 10.1038/s41586-021-03363-z PMC8046668 33762734

